# The Effect of *C. burnetii* Infection on the Cytokine Response of PBMCs from Pregnant Goats

**DOI:** 10.1371/journal.pone.0109283

**Published:** 2014-10-03

**Authors:** Anne Ammerdorffer, Hendrik-I J. Roest, Annemieke Dinkla, Jacob Post, Teske Schoffelen, Marcel van Deuren, Tom Sprong, Johanna M. Rebel

**Affiliations:** 1 Department of Medicine, Radboud University Medical Center, Nijmegen, The Netherlands; 2 Department of Bacteriology and TSEs, Central Veterinary Institute of Wageningen UR, Lelystad, The Netherlands; 3 Department of Infection Biology, Central Veterinary Institute of Wageningen UR, Lelystad, The Netherlands; 4 Department of Internal Medicine, Canisius-Wilhelmina Hospital, Nijmegen, The Netherlands; 5 Department of Medical Microbiology and Infectious Diseases, Canisius- Wilhelmina Hospital, Nijmegen, The Netherlands; Texas A&M Health Science Center, United States of America

## Abstract

In humans, infection with *Coxiella burnetii*, the causative agent of Q fever, leads to acute or chronic infection, both associated with specific clinical symptoms. In contrast, no symptoms are observed in goats during *C. burnetii* infection, although infection of the placenta eventually leads to premature delivery, stillbirth and abortion. It is unknown whether these differences in clinical outcome are due to the early immune responses of the goats. Therefore, peripheral blood mononuclear cells (PBMCs) were isolated from pregnant goats. In total, 17 goats were included in the study. Six goats remained naive, while eleven goats were infected with *C. burnetii*. Toll-like receptor (TLR) and cytokine mRNA expression were measured after in vitro stimulation with heat-killed *C. burnetii* at different time points (prior infection, day 7, 35 and 56 after infection). In naive goats an increased expression of interleukin (IL)-1β, tumor necrosis factor (TNF)-α, IL-10 and interferon (IFN)-γ mRNA upon *C. burnetii* stimulation was detected. In addition, TLR2 expression was strongly up-regulated. In goats infected with *C. burnetii*, PBMCs re-stimulated in vitro with *C. burnetii*, expressed significantly more TNF-α mRNA and IFN-γ mRNA compared to naive goats. In contrast, IL-10 mRNA production capacity was down-regulated during *C. burnetii* infection. Interestingly, at day 7 after inoculation a decreased IFN-γ protein level was observed in stimulated leukocytes in whole blood from infected goats, whereas at other time-points increased production of IFN-γ protein was seen. Our study shows that goats initiate a robust pro-inflammatory immune response against *C. burnetii* in vitro. Furthermore, PBMCs from *C. burnetii* infected goats show augmented pro-inflammatory cytokine responses compared to PBMCs from non-infected goats. However, despite this pro-inflammatory response, goats are not capable of clearing the *C. burnetii* infection.

## Introduction

Q fever is a zoonosis caused by the intracellular Gram-negative bacterium *Coxiella burnetii*. In humans, infection may remain unnoticed in approximately half of the infected individuals and causes disease, a short flu-like acute febrile disease, pneumonia or hepatitis, in the other half [1]. After infection, either unrecognized or symptomatic, 1 to 5% may develop chronic Q fever months to years later, which presents primarily as endocarditis or vascular infection and may be lethal when left untreated [1]. Infected goats, sheep and cattle are the main reservoir for human infections, these animals can excrete high numbers of *C. burnetii* into the environment during parturition [2], [3].

In contrast to human infections, infection in goats by *C. burnetii* does not lead to recognizable systemic disease in non-pregnant animals, while in pregnant animals infection will lead only to abortion, stillbirth or premature delivery explained by the high bacterial numbers in the placenta and severe local inflammation [4]. Although clinical symptoms are absent in goats, two weeks after infection a clear specific humoral immune response occurs with the appearance of IgM and IgG anti-Phase 2 *C. burnetii* antibodies [3]. So far little is known about the early immune response in goats and the differences in the immune response to *C. burnetii* in man and goat.

Toll-like receptors (TLRs) are assumed to be the principal receptors involved in the early recognition and signalling of *C. burnetii* by innate immune cells. Upon recognition, peripheral blood mononuclear cells (PBMCs) derived from healthy humans produce various cytokines including interleukin-1beta (IL-1β), tumour necrosis factor-alpha (TNF-α) and interleukin-10 (IL-10), but no interferon gamma (IFN-γ) [5]–[7]. After infection, stimulation of human PBMCs with *C. burnetii* also leads to up-regulation of IL-1β [6], [8]–[10]. In patients with chronic Q-fever, the anti-inflammatory cytokine IL-10 is up-regulated [7], [11].

It is generally assumed that the type and degree of the early innate immune response relates to the severity of early disease manifestations and determines the early outgrowth of the infection [12]. Therefore it may be hypothesized that this response is different in goats which do not develop disease after infection as compared to humans. To test this hypothesis we evaluated the *C. burnetii* induced TLR and cytokine responses of PBMCs derived from naive and from infected pregnant goats in the last months of pregnancy. Furthermore, Roest et al. recently examined the circulating cytokine levels in naive and *C. burnetii* infected goats. They observed an absence of a systemic cytokine mRNA response and hypothesized that PBMCs might not have been in contact with *C. burnetii* during infection. To clarify this we focus on PBMCs and their ability to induce an immune response against heat-killed *C. burnetii*.

## Material and Methods

### Animals, Infection and Experimental Design

Seventeen pregnant healthy Alpine yearling goats were included in the study. The experimental setup has been published in detail by Roest et al. [3]. In short, on day 76 of the pregnancy eleven goats were intranasally inoculated with 1 ml of 1×10∧6/ml *C. burnetii* X09003262-001 (*C. burnetii* 3262) in culture medium. As a negative control,six goats were inoculated with 1 ml of culture medium only. Serum samples were taken weekly to measure antibody titers against *C. burnetii* (LSIVET RUMINANT milk/serum Q-fever ELISA kit, LSI, France). EDTA and heparin blood samples for stimulation experiments were taken from each goat; at day 0 (before inoculation) and at day 7, 35 and 56. To prove successful *C. burnetii* infection of the infected goats, the placenta was sampled after parturition from each goat for immunohistochemistry (IHC) and vaginal mucus was sampled for detection of *C. burnetii* DNA by PCR.

### Ethics Statement

All animal experiments were approved by the Animal Experiment Commission of the Central Veterinary Institute, part of Wageningen UR, in accordance with the Dutch regulations on animal experimentation (registration number 2011111.c). Everything possible was done to minimise animal suffering. Humane endpoints were defined in advance. Whenever these endpoints were reached, animals were euthanized.

### Coxiella burnetii strains

*C. burnetii* 3262 was used as infectious organism. This strain was isolated from the placenta from a goat that aborted on a farm during the Q fever outbreak in the Netherlands [13]. The strain was genotyped as CbNL01, which was the main *C. burnetii* genotype during the Dutch Q fever outbreak [13]. Ex vivo stimulation experiments were performed with the heat-killed *C. burnetii* Nine Mile (NM) reference strain. Both *C. burnetii* NM and *C. burnetii* 3262 were cultured at the Central Veterinary Institute as described previously [3]. The strains represent LPS phase I. LPS phase determination was performed by SDS-PAGE and silver staining, using purified phase I (RSA493) and phase II (RSA439) *C. burnetii* NM LPS (kindly provided by R. Toman) as controls [14], [15]. The number of *Coxiella* DNA copies was determined using Taqman (Quanta BioSciences, USA) real-time PCR as mentioned earlier [4]. MLVA typing was performed by using 12 of the 17 loci described by Arricau-Bouvery et al. [16]. A second series of stimulation experiments was simultaneously performed also with three heat-killed isolates, *C. burnetii* 14160-001, *C. burnetii* 3345937 and *C. burnetii* 3262. Details of all strains are mentioned in Table 1. Bacteria were killed by heating for 30 minutes at 99°C.

**Table 1:** List of *C. burnetii* isolates used in this study.

Name	Country of origin	Source	LPS Phase	MLVA marker and number of repeats											
				01	03	20	21	22	28	24	30	31	34	27	36
*C. burnetii* 3262	The Netherlands	Goat placenta	I	4	7	19	6	6	3	11	5	3	7	3	13
*C. burnetii* 3345937	The Netherlands	Human heart valve	I	4	7	19	6	6	3	11	5	3	7	3	13
*C. burnetii* 14160-001	The Netherlands	Goat placenta	I	3	6	15	6	6	7	13	6	3	9	2	4
*C. burnetii* Nine Mile	USA	Tick	I	4	7	15	6	6	6	27	6	5	5	4	4

### Isolation and stimulation of peripheral blood mononuclear cells

The PBMC fraction was obtained by density centrifugation of EDTA blood using a Ficoll-Paque gradient (Pharmacia Biotech, USA) or Leucosep tubes prefilled with Ficoll-Paque (Greiner, the Netherlands). After washing, the cells were re-suspended in RPMI 1640 Dutch modified (Gibco Invitrogen, USA) supplemented with 50 mg/L gentamycin (Centrafarm, the Netherlands), 2 mM L-glutamine, and 1 mM pyruvate (Gibco Invitrogen, USA). A volume of 100 µl containing 5×10^5^ mononuclear cells was added to round-bottom 96-well plates (Costar, Corning, The Netherlands) and incubated at 37°C and 5% CO_2_ for both 4 h and 24 h with either 50 µl RPMI (medium control), *C. burnetii* NM 1×10^7^/ml, *C. burnetii* 3262 1×10^6^/ml, *C. burnetii* 3345937 3.2×10^4^/ml, *C. burnetii* 14160-001 1×10^7^/ml and *E. coli* LPS 10 ng/ml in the presence of 50 µl 5% naive goat serum (in house). Following incubation, the supernatant was harvested and stored at −20°C. Subsequently Trizol (Invitrogen, USA) was added to cell pellet and stored at −80°C till further RNA isolation.

### mRNA expression of Toll-like receptors and cytokines

RNA was isolated from the Trizol samples using the manufacturer's guidelines. In the end, the pellet was re-suspended in 25 µl RNAse free water (Sigma–Aldrich, USA) with 0.5 µl RNAseOUT Recombinant ribonuclease Inhibitor (Invitrogen, USA). Further purification of the samples was performed with the DNA-free kit (Ambion, USA) and the concentration of the RNA samples was determined using the Nanodrop (ND1000, NanoDrop Technologies, Inc, USA). Reverse transcription was performed using random primers and SuperScript III Reverse Transcriptase (Invitrogen, USA). PCR on the cytokines mRNA (TNF-α, IL-1β, IL-10 and IFN-γ) was performed using specific goat primers [3] and SYBR Green PCR Master Mix (Applied Biosystems, USA) in an ABI 7500 Real-time PCR system (PE Applied Biosystems, USA). Results were normalized according to the Succinate dehydrogenase complex subunit A (SDHA) gene of the same sample (Bos Taurus, Primerdesign Ltd, United Kingdom). Data was not corrected for the medium control, hereby the influences of the pregnancy on the cytokine mRNA expression remain visible. The primer sets for TLR1, TLR2, TLR4 and TLR6 were designed using PrimerExpress (Invitrogen, USA) (Table 2). The Livak method (2∧ΔΔCt) was used to represent the TLR expression data.

**Table 2:** Primer sequences for TLR1, TLR2, TLR4 and TLR6.

Name	Primer sequence 5′-3′ Forward/reverse	Accession no.	Annealing Temperature
TLR1	GGGTTGAGTGCCACACAGTTACCCCATAAGTATCTCCTAAGACCAATAAAA	HQ263209.1	60°C
TLR2	ACTGGGTGGAGAACCTCATGGTCATTTGCCAGGGACGAAGTC	HQ263214.1	60°C
TLR4	TCTGCCTTCACTACAGGGACTTTATGGCTCTTGTGGAAACCTTCCT	HM627213.2	60°C
TLR6	TCTCAAGCATTTAGACCTCTCATTCACTGGGTCAAGTTGCCAAATTC	HQ263211.1	60°C

### Whole blood stimulation

Undiluted heparin blood (500 µl) was stimulated with either nothing or 25 µl heat-killed *C. burnetii* NM 1×10^7^/ml. As control, samples were stimulated with concanavalin A (ConA) and/or phytohemagglutinine (PHA). After 24 h incubation at 37°C, the samples were centrifuged and the supernatant was stored at −20°C till further investigation. The production of IFN-γ protein by the leukocytes in the whole blood samples was analysed with the Bovigam IFN-γ test kit for cattle (Prionics, Schileren-Zurich, Switzerland) which was evaluated for goats. ELISA results were obtained as Optical Density determined at 450 nm (OD_450_) with a 635 nm reference filter (EL 808 Ultra micro plate reader, Bio-tek instruments, USA). Recombinant goat IFN-γ was purchased from Cusabio to invest to proportion between the measured OD values and the IFN-γ concentration. Hereto, a dilution series of IFN-γ was made with a maximum concentration of 25 ng/ml. The IFN-γ concentration and the corresponding OD values are proportional. Because the Bovigam IFN-γ test kit is able to measure goat IFN-γ in a dose-dependent manner, we decided to use this kit in our study.

### Statistical analysis

Statistics regarding mRNA expression and cytokine production were performed using GraphPad Prism 5 software (Graphpad). Differences between experimental groups were tested using the Mann-Whitney *U* test, and differences with a *p*-value ≤0.05 were considered statistically significant. Samples with values lower or higher than two times the standard deviation were defined as outliers and excluded from the statistical analysis. These outliers are indicated in the figures as an open circle. In case the value of the outlier exceeded the y-axis of the graph, its value is mentioned. The PCR efficiency was determined for all TLRs and SDHA using a standard curve generated with different dilutions of a mixture of the cDNA of all samples.

## Results

### Experimental *C. burnetii* infection in goats

The experimental infection of the goats with *C. burnetii* 3262 was successful in all infected goats as shown by a positive IHC of the cotyledon with adjacent allantochorion of the placenta and by positive PCR of vaginal swabs. All naive goats remained negative in both tests. The IHC pictures of a *C. burnetii* positive and negative cotyledon can be found in the article of Roest et al.[4] in which the goats from experiment III resemble the goats in our study. Three infected goats gave birth to dead lambs, the lambs of the remaining six infected goats were alive. The offspring of the naive goats were all alive and healthy.

**Figure 1:**
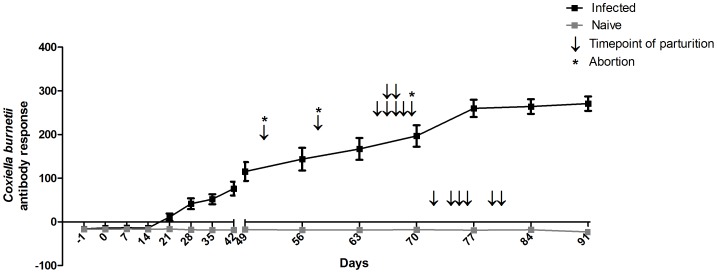
Successful experimental infection of goats with *Coxiella burnetii* 3262. Eleven out of seventeen goats were intranasally infected with *C. burnetii* 3262. The remaining six were kept naive. During the infection, antibodies against *C. burnetii* were measured weekly in the serum of all goats using the LSIVET RUMINANT milk/serum Q-fever ELISA kit. Grey squares represent naive goats, black squares represent infected goats. Median values with SEM are shown. Parturition is indicated for each individual goat using an arrow. Abortion, defined as birth of dead lambs, is indicated with an asterisk. The final two remaining pregnant infected goats were culled for other purposes, and therefore not included in this graph.

The weekly antibody response of all goats against *C. burnetii* during the infection period is shown in Figure 1. The antibody response in the infected goats increased strongly 21 days after infection, whereas the antibody response of the naive goats did not alter. The average pregnancy of the infected goats was 143 days (range 128–146 days), significantly shorter (p<0.001) than the 153 days (range 149–157 days) of the naive goats.

### TLR up-regulation and cytokine responses in *C. burnetii* stimulated PBMCs from all goats at day 0

**Figure 2:**
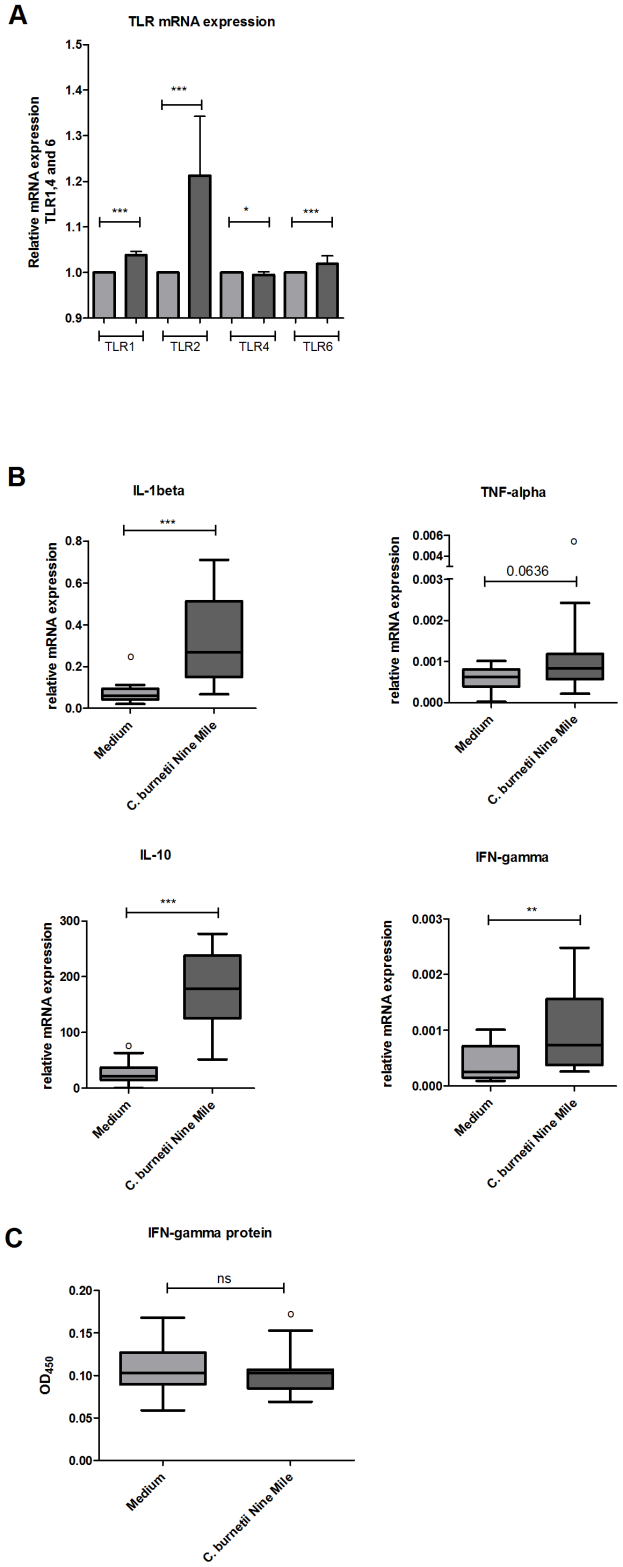
TLR up-regulation and cytokine responses of *C. burnetii* stimulated PBMCs from naive goats. PBMCs were isolated from EDTA blood sampled from 17 goats prior to inoculation. **2A/B**: PBMCs were incubated 4 h (TLRs, IL-1β, TNF-α) or 24 h (IL-10, IFN-γ) with either medium (grey bars) or heat killed *C. burnetii* NM 1×10^7^/ml (dark grey bars). mRNA expression of TLR1, 2, 4, 6 and IL-1β, TNF-α, IL-10 and IFN-γ was determined using qPCR. **2C**: IFN-γ protein was measured in the supernatant of PBMCs stimulated for 24 h with nil or *C. burnetii* NM 1×10^7^/ml. IFN-γ was measured using the Bovigam Elisa kit. Box and Whisker plots are shown. Outliers are indicated as open circles. NS not significant; *p<0.05; **p<0.01; ***p<0.001, Mann-Whitney U-test.

Stimulation of PBMCs from all pregnant goats at day 0 (prior to infection) with *C. burnetii* NM for 4 hours resulted in a slightly lower mRNA expression of TLR4, the mRNA expression of TLR1 and TLR6 was slightly up-regulated, and the expression of TLR2 mRNA was strongly up-regulated in comparison with unstimulated PBMCs (Figure 2A). In general, a fold induction of factor 2 indicates biological relevance. Despite the significantly increased TLR1, TLR2 and TLR6 mRNA expression after *C. burnetii* stimulation, the fold induction remained below factor 2. The mRNA expression of IL-1β and IFN-γ was up-regulated after stimulation of PBMCs from naive goats with the positive control *E. coli* LPS (data not shown), showing the reliability of the experimental approach to measure cytokine mRNA expression in goat PBMCs. Stimulation of goat PBMCs with *C. burnetii* NM resulted in an increased mRNA expression of IL-1β and the same trend was observed for TNF-α (Figure 2B). In PBMCs stimulated for 24 hours, the expression of the anti-inflammatory cytokine IL-10 strongly increased. Furthermore, the mRNA expression of the T-cell or NK-cell derived cytokine IFN-γ also showed an increase (Figure 2B), although this did not lead to increased IFN-γ protein concentrations in the supernatants of these cells (Figure 2C).

### PBMC stimulation experiments from naive and infected pregnant goats during the course of pregnancy

**Figure 3:**
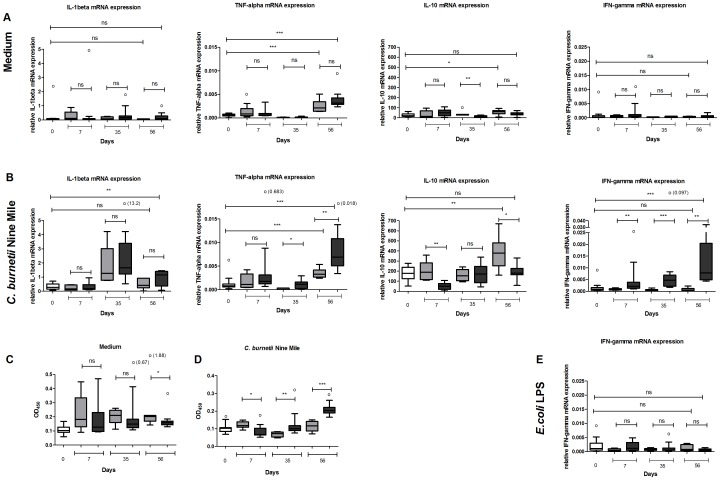
Cytokine profile of *C. burnetii* stimulated PBMCs from naive and *C. burnetii* infected goats. **3A/B:** EDTA blood was sampled at day 7, 35 and 56 of the study from six naive goats (grey bars) and eleven infected goats (dark grey bars). The white bars show the mRNA expression at day zero. PBMCs were isolated and incubated for 4 h or 24 h with either medium (**3A**), heat killed *C. burnetii* NM 1×10^7^/ml (**3B**) or *E. coli* LPS 10 ng/ml (**3E**). mRNA expression of TNF-α, IL-1β, IL-10 and IFN-γ was determined using qPCR. **3C/D**: IFN-γ protein was measured in the supernatant of PBMCs stimulated for 24 h with nil (**3C**) or *C. burnetii* NM 1×10^7^/ml (**3D**). IFN-γ was measured using the Bovigam Elisa kit. Grey bars represent six naive goats, dark grey bars represent eleven infected goats. Box and Whisker plots are shown. Outliers are indicated as open circles. NS; not significant *p<0.05; **p<0.01; ***p<0.001, Mann-Whitney U-test.

The course of the cytokine mRNA expression of PBMCs was investigated in unstimulated PBMCs, derived from naive and infected goats during the study period of 56 days (Figure 3A). IL-1β mRNA was not up-regulated in PBMCs from both naive and infected pregnant goats. At day 56 of the study TNF-α mRNA expression was increased in PBMCs derived from both naive and infected goats compared to TNF-α mRNA expression at the start of the study. In the infected goats this increase of TNF-α mRNA was significantly higher than in non-infected goats. In contrast, the IL-10 mRNA expression at day 56 was increased in the PBMCs from non-infected goats, while this increase was not observed in the PBMCs of infected goats. Both IFN-γ mRNA and IFN-γ protein levels were not up-regulated in respectively unstimulated PBMCs and unstimulated whole blood from both naive and infected pregnant goats during the study period (Figure 3A and 3C).

To investigate whether stimulation with *C. burnetii* resulted in different TLR up-regulation and cytokine responses of PBMCs derived from naive or infected goats we stimulated these cells in vitro with *C. burnetii* NM. The mRNA expression of TLR1, TLR2, TLR4 and TLR6 of *C. burnetii* NM stimulated PBMCs derived from naive and infected goats at day 35 equalled the expression as prior infection (data not shown). The expression of IL-1β and TNF-α mRNA of the infected goats was significantly higher at day 56 of the study than at the start of the study (Figure 3B). In contrast, *C. burnetii* stimulated PBMCs from naive goats did not show an increased IL-1β and IFN-γ mRNA expression. The expression of TNF-α mRNA at day 56 of the study was significantly higher in the infected goats compared to the naive goats. Compared to day zero, IL-10 mRNA expression increased within the 56 days of the study in the naive goats. In contrast, no significant difference in IL-10 mRNA expression between day zero and day 56 was observed in the infected goats. In addition, the expression of IL-10 mRNA was lower in the infected goats compared with the naive goats at day 7. The infected goats expressed more IFN-γ mRNA after PBMC stimulation starting from day 7 onwards (Figure 3B). Furthermore, at day 56 we found that in infected goats IFN-γ protein in the supernatants was higher compared to naive goats (Figure 3D) and unstimulated PBMCs (P<0.0017). At other time points, IFN-γ protein in the *C. burnetii* stimulated samples did not exceed that of the unstimulated samples, but the IFN-γ protein level was significantly lower in the PBMCs obtained of infected goats compared to PBMCs of naive goats at day 7 and higher at day 35. The increased IFN-γ mRNA expression found in the infected goats at day 35 and 56 after *C. burnetii* NM stimulation was not observed when PBMCs of these infected goats were stimulated with *E. coli* LPS (Figure 3E).

### Whole blood stimulation experiments from naive and infected pregnant goats during the course of pregnancy

**Figure 4:**
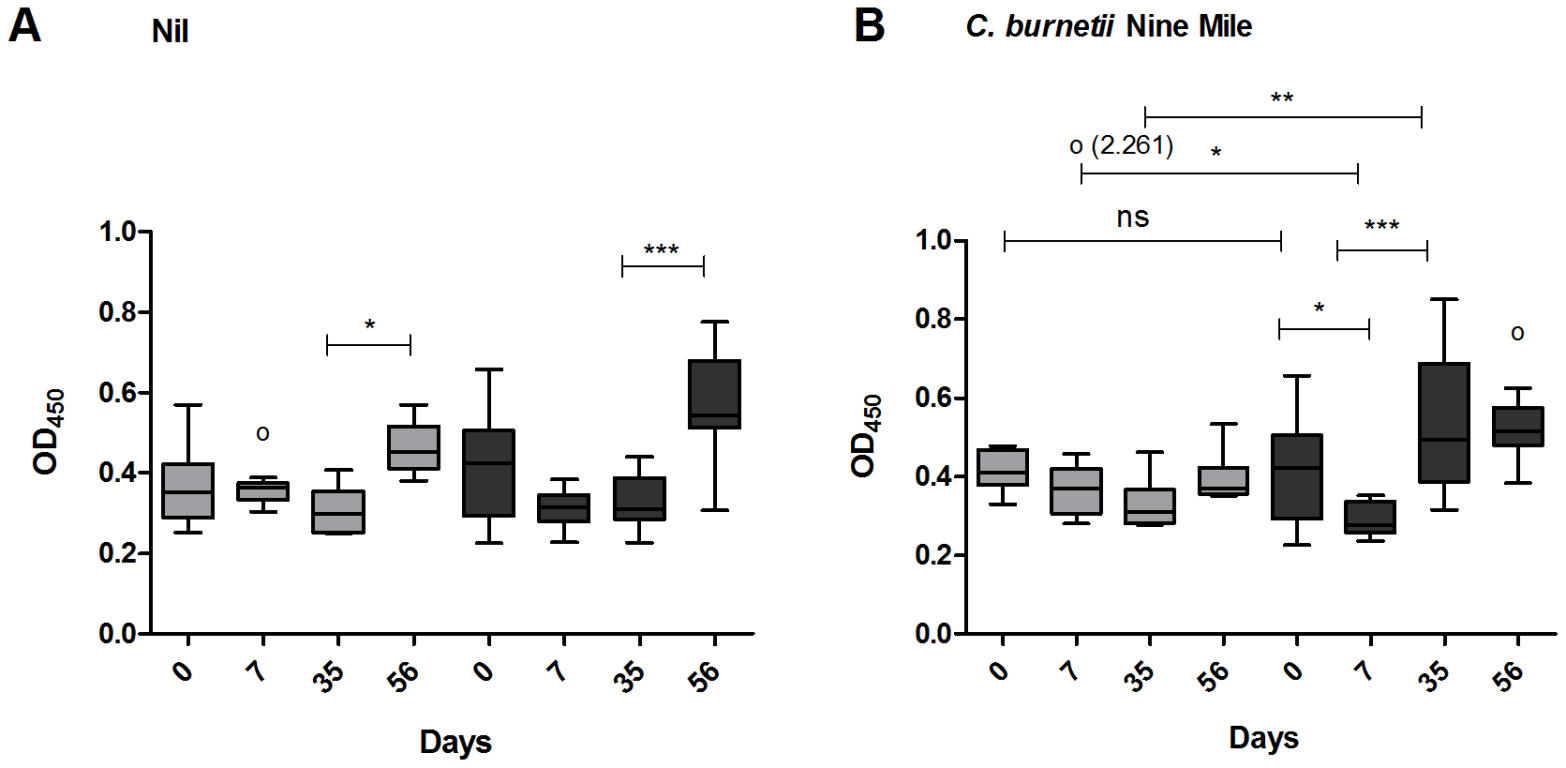
Increased Interferon-γ production in *C. burnetii* infected goats after stimulation with *C. burnetii*. Stimulation of undiluted heparin blood with nil (4A) or heat killed *C. burnetii* NM 1×10^7^/ml (4B). Grey bars represent six naive goats, dark grey bars represent eleven infected goats. Blood was sampled at day zero and at day 7, 35 and 56 of the study. IFN-γ was measured after 24 h incubation using the Bovigam IFN-γ ELISA kit. Box and Whisker plots are shown. Outliers are indicated as open circles. NS; not significant *p<0.05; **p<0.01; ***p<0.001, Mann-Whitney U-test.

The role of whole blood components on the early immune response against *C. burnetii* was investigated by stimulation of whole blood from naive and infected goats with nil or *C. burnetii* NM. In non-stimulated blood from both naive and infected goats, IFN-γ production did not significantly alter at day 7 and day 35 of the study. However, at day 56, IFN-γ concentration increased in both naive as infected goats (Figure 4A). Figure 4B shows that in vitro stimulation of whole blood with *C. burnetii* NM does not lead to changes in the IFN-γ production in naive goats during the 56 days. In whole blood of the infected goats, the IFN-γ protein level is decreased at day 7 which was not observed in the naive goats. At day 35 of the study, the IFN-γ level is significantly higher in whole blood of infected goats compared to naive goats after stimulation with *C. burnetii* NM.

### In vitro stimulation of PBMCs from naive and infected pregnant goats with different *C. burnetii* strains

**Figure 5:**
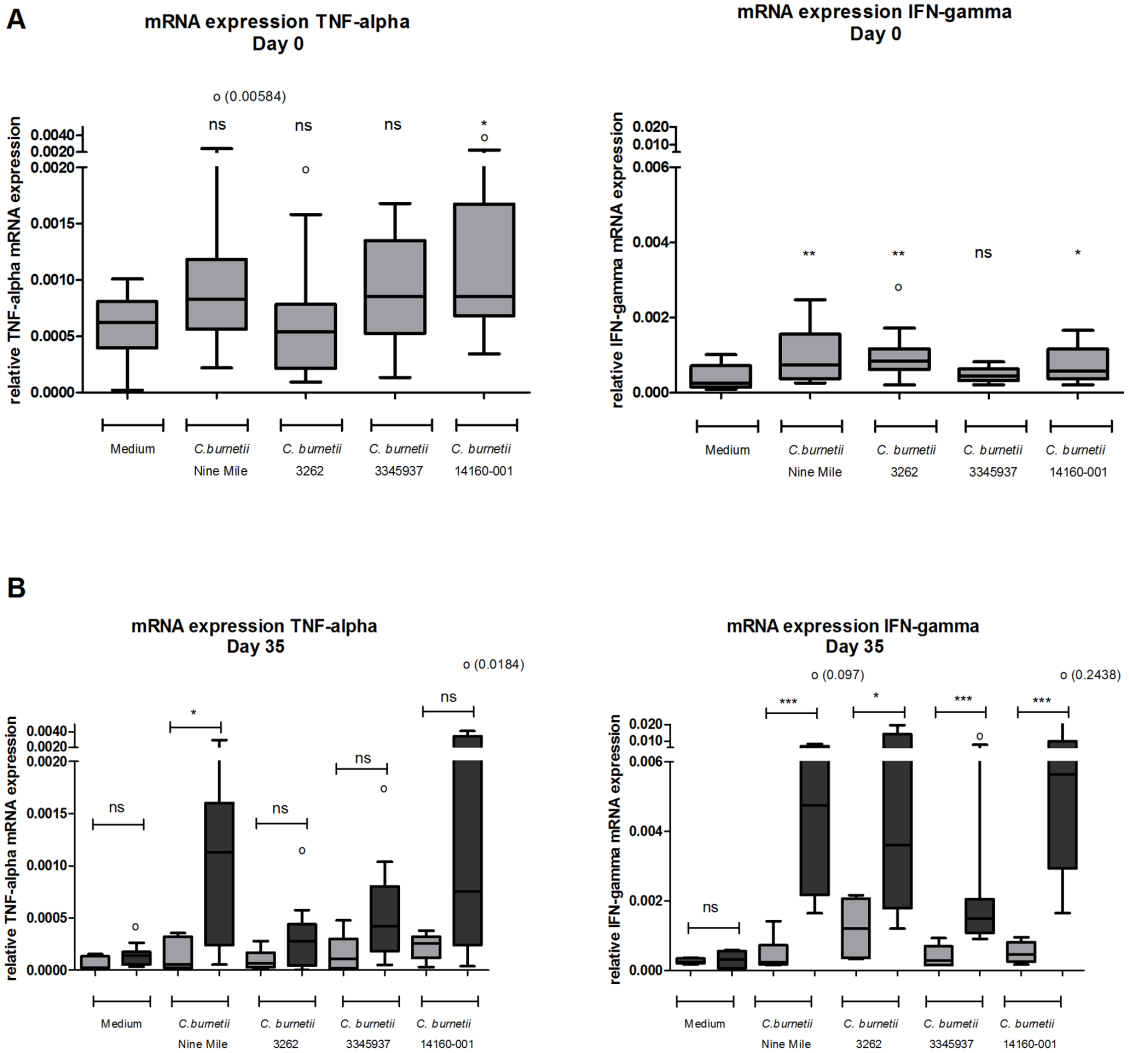
Stimulation of *C. burnetii* infected PBMCs results in an increased cytokine expression. EDTA blood was sampled at day zero (5A) and day 35 (5B) of the study. Peripheral blood mononuclear cells were isolated and incubated for 4 h or 24 h with either medium, heat killed *C. burnetii* NM 1×10^7^/ml, heat killed *C. burnetii* 3262 1×10^6^/ml, heat killed *C. burnetii* 3345937 3.2×10^4^/ml, heat killed *C. burnetii* 14160-001 1×10^7^/ml. mRNA expression of TNF-α and IFN-γ was determined using qPCR. Grey bars represent six naive goats, dark grey bars represent eleven infected goats. Box and Whisker plots are shown. Outliers are indicated as open circles. NS not significant; *p<0.05; **p<0.01; ***p<0.001; ****p<0.0001, Mann-Whitney U-test.

PBMCs from naive and infected goats were stimulated in vitro with four different *C. burnetii* strains to investigate whether all strains were able to induce up-regulation of cytokine mRNA levels. At day zero, only *C. burnetii* 14160-001 induced significantly more TNF-α mRNA compared to unstimulated PBMCs. The IFN-γ mRNA levels increased after PBMC stimulation with *C. burnetii* NM, *C. burnetii* 3262 and *C. burnetii* 14160-001. *C. burnetii* 3345937 stimulation did not induce either TNF-α and IFN-γ mRNA (Figure 5A). At day 35, PBMCs re-stimulated with all strains, except *C. burnetii* 3262, showed a trend towards an increased TNF-α mRNA expression in PBMCs of infected goats than PBMCs of naive goats. IFN-γ mRNA expression was significantly increased in PBMCs from infected goats after encounter with all four *C. burnetii* strains compared to naive goats (Figure 5B).

## Discussion

PBMCs from pregnant goats have a clear cytokine response upon contact with *C. burnetii*. This response is observed both in goats without any previous contact with the bacterium as well as in goats who where recently infected. This latter observation is interesting because infections of pregnant goats do not lead to systemic disease of the animal except for stillbirth of the lamb. In PBMCs of healthy pregnant goats we observed that the mRNA expression of IL1-β, TNF-α, IL-10 and IFN-γ were up-regulated after *C. burnetii* stimulation. These findings correspond with other studies in which increased TNF-α protein levels were found in human monocyte-derived dendritic cells stimulated with the *C. burnetii* outer membrane protein 1, and THP-1 cells which showed increased TNF-α and IFN-γ expression after *C. burnetii* stimulation [17], [18]. Recently, Graham et al.[19] demonstrated that stimulation of human alveolar macrophages with *C. burnetii* skewed towards a Th1 response. Increased TNF-α and IFN-γ protein levels were also found in serum from mice challenged with *C. burnetii*, and TNF-α mRNA expression increased in mice of which bone marrow derived macrophages were stimulated with *C. burnetii* [20], [21]. Thus, our results show that PBMCs of goats do not differ from humans and mice in their augmented TNF-α and IFN-γ mRNA response after in vitro stimulation with *C. burnetii*. During *C. burnetii* infection increased IFN-γ protein was measured in *C. burnetii* stimulated whole blood of goats which corresponds with the higher IFN-γ protein levels found in infected mice and chronic Q fever patients [20], [22].

The expression of IL-1β, IL-10 and IFN-γ mRNA was not up-regulated in the PBMCs of pregnant goats who were infected with *C. burnetii*. The observed up-regulation of TNF-α mRNA expression in PBMCs derived from infected goats may be seen as a consequence of pregnancy because this pattern was also found in PBMCs from naive goats. A possible explanation for the absence of an increased cytokine mRNA expression is that the non re-stimulated PBMCs did not respond to a nasal infection of *C. burnetii*. One of the reasons can be that in goats the *C. burnetii* infection is localised specifically in the placenta, and therefore no persistent systemic mRNA up-regulation was observed in the PBMCs from infected goats. Ben Amara et al.[23] indeed found that *C. burnetii* replicates in vitro in placental derived trophoblasts and induces an up-regulation of cytokine mRNA. Therefore, they assumed that the specific inflammatory environment in the placenta, skewed towards tolerance and anti-inflammatory responses, permits persistence of *C. burnetii*. This specific compartmentalized infection is also observed in chronic Q fever patients, as in these persons *C. burnetii* is mainly localised in the heart valve and vessels [1].

At day 56 of our study TNF-α mRNA expression was increased in all goats compared to the initial level, but a significantly higher TNF-α mRNA expression was seen in the infected goats. This suggests that, besides the influence of the pregnancy, infection with *C. burnetii* also increases expression of TNF-α mRNA. We also showed a clear increase in IL-1β and IFN-γ mRNA expression in the *C. burnetii* NM stimulated PBMCs derived from infected goats from day zero till day 56 of the study. However, at day 56, only IFN-γ mRNA expression was significantly higher in the PBMCs from infected goats. The fold induction (*C. burnetii* stimulated PBMCs divided by unstimulated PBMCs) of naive and infected goats at day 56 was respectively 1.96 and 24.21, indicating the large effect on IFN-γ mRNA expression of *C. burnetii* stimulated PBMCs from infected goats.

The fold induction at day 56 in *C. burnetii* stimulated PBMCs from naive and goats was respectively 1.96 and 24.21. Besides the observed mRNA up-regulation, IFN-γ protein levels were increased in *C. burnetii* stimulated PBMCs and whole blood from infected goats compared to naive goats. Thus although no systemic response in goats could be observed, PBMCs of *C. burnetii* infected goats respond differentially to *C. burnetii* stimulation compared to PBMC of uninfected goats. Stimulation with *E. coli* LPS did not lead to increased up-regulation of IFN-γ mRNA expression in the *C. burnetii* infected goats, which demonstrates that the observed IFN-γ response is specific for *C. burnetii*. Even though we hypothesized that *C. burnetii* infected PBMCs are translocated to the placenta, we assume that remaining memory T-cells in the peripheral blood can be activated and play a role in this immune response and increased IFN-γ mRNA expression.

The importance of TNF-α and IFN-γ during *C. burnetii* infection has been shown by Andoh et al. [24]. These authors demonstrated that IFN-γ^−/−^ and TNF-α^−/−^ mice have respectively high and modest susceptibility to *C. burnetii* NM infection and that the disease progressed rapidly in these mice [24]. In humans, much research has been performed on the cytokine expression in chronic Q fever patients, a condition that in our opinion mostly resembles the *C. burnetii* re-stimulation of PBMCs from infected goats. TNF-α mRNA and TNF-α protein levels are increased in *C. burnetii* re-stimulated monocytes of chronic Q fever patients compared to healthy controls [9]. Similarly, blood cells of chronic Q fever patients respond to *C. burnetii* stimulation with higher IFN-γ production [10], [22].

The observed up-regulation of IFN-γ mRNA expression after *C. burnetii* NM stimulation was also found for the inoculum strain *C. burnetii* 3262 and the other *C. burnetii* strains which indicates that the different *C. burnetii* strains contain corresponding antigens on their surfaces which are recognized by goat PBMCs. Similar cross reactivity was demonstrated by Arricau-Bouvery et al. in a vaccination study in goats [25]. Based on these results we decided to perform our stimulation experiments with *C. burnetii* Nine Mile. The concentrations of the different *C. burnetii* strains used in this experiment were not identical, due to time restrictions and culture difficulties. Therefore, it is not possible to compare the strains and the cytokine mRNA expression they induce. Further studies have to be performed to investigate the possible divers immune response against *C. burnetii* strains by goats.

PBMCs derived from goats infected with *C. burnetii* expressed significantly less or equal amounts of IL-10 mRNA compared to the PBMCs of non-infected goats. We did not expect this, as in humans high IL-10 protein levels are related to chronic Q fever infection, and in acute Q fever patients a slight increase of IL-10 production was observed compared to healthy controls [7], [11]. The lower expression of IL-10 mRNA in the infected goats can be explained by the higher levels of IFN-γ mRNA and IFN-γ protein, as these two cytokines have an antagonistic effect on each other. Our results indicate that PBMCs from infected goats actually did interact with *C. burnetii*, leading to an augmented cytokine mRNA expression after a second *C. burnetii* encounter. The amount of IL-10 mRNA exceeded the mRNA levels of the housekeeping gene much more (up to 400 fold) than for IL-β and IFN-γ. However, the fold induction of IL-10 (*C. burnetii* stimulated PBMCs divided by unstimulated PBMCs) did not differ from the IL-1β fold induction or was even lower in the infected goats at day 35 and 56 when compared with IFN-γ. For example, at day 56, the fold induction of IL-1β, IL-10 and IFN-γ in the infected goats was respectively 5.89, 5.04 and 24.21.

We can speculate that regarding the pro-inflammatory cytokines, TNF-α and IFN-γ, PBMCs of infected goats re-stimulated with *C. burnetii* show the same cytokine profile as chronic Q fever patients, while the IL-10 mRNA expression in infected goats is not up-regulated as found in the chronic Q fever patients. These differences in IL-10 expression could be an explanation for the divergent clinical outcomes in humans and goats during *C. burnetii* infection. Yet, is should be taken in to account that, in contrast with the human study subjects, the infected goats are pregnant which can influence the cytokine responses in general and upon encounter of micro-organisms. Furthermore, possible differences in experimental approaches should be considered, i.e. our conclusions are based on mRNA expression levels, while other studies rely on protein levels in particular.

An interesting finding is the decreased IFN-γ protein level in *C. burnetii* stimulated PBMCs and whole blood from infected goats at day 7 of the study. This reduction was not observed in the PBMCs and whole blood of naive goats and is inconsistent with the increased IFN-γ protein levels observed in the PBMCs obtained from infected goats later in the infection. It can be hypothesized that the initial *C. burnetii* infection in the goats is capable to immune modulate PBMCs to produce less IFN-γ. Other microorganism infections, like *Legionella pneumophila*, *Leishmania* and *Mycobacterium tuberculosis* are also capable of modulating the Th1 response during early phases of infection [26]–[28]. Modulation of the IFN-γ response early during the infection, could create a favorable situation for *C. burnetii* to escape the early immune response of the goats, and might therefore be a strategy to pass over to the placenta without being eliminated. PBMCs obtained from infected goats later in the infection did not show the IFN-γ decrease after re-stimulation in vitro. This could mean that the immune modulation of PBMCs in vivo only occurred early in infection and once the *Coxiella* bacteria have reached the placenta, no systemic immune modulation is needed, or all systemic *Coxiella* bacteria are eliminated. Antigen specific lymphocyte sequestration can also be an explanation for the lower IFN-γ levels in infected goats up to 7 days after the infection. However, our results suggests that the effector T-cell population (Th1) remained in the PBMC fraction during *Coxiella* infection, as the mRNA expression of IFN-γ increased. However, based on the results of IL-10 mRNA expression, we can hypothesize that the regulatory T-cells (Th2) could be transferred to the lymph nodes during *Coxiella* infection.

During first contact with *C. burnetii*, PBMCs of healthy pregnant goats are capable of recognizing *C. burnetii* and activate their early immune response in vitro. Furthermore, PBMCs from infected goats showed an augmented pro-inflammatory cytokine mRNA expression compared to PBMCs of naive goats after *C. burnetii* stimulation, whereas the anti-inflammatory cytokine IL-10 was down-regulated. The finding that PBMCs from goats that have previously had contact with *C. burnetii* react differently than naive PBMCs can be useful in future vaccine strategies. Finally, although the PBMC response as found in infected goats is strongly geared towards a pro-inflammatory state, the infection is not cleared and the goats will still suffer abortions and stillbirths.
